# Prediction Models for Sleep Quality Among College Students During the COVID-19 Outbreak: Cross-sectional Study Based on the Internet New Media

**DOI:** 10.2196/45721

**Published:** 2023-03-24

**Authors:** Wanyu Zheng, Qingquan Chen, Ling Yao, Jiajing Zhuang, Jiewei Huang, Yiming Hu, Shaoyang Yu, Tebin Chen, Nan Wei, Yifu Zeng, Yixiang Zhang, Chunmei Fan, Youjuan Wang

**Affiliations:** 1 The Second Affiliated Hospital of Fujian Medical University Quanzhou China; 2 The School of Public Health, Fujian Medical University Fuzhou China; 3 The Graduate School of Fujian Medical University Fuzhou China; 4 The School of Clinical Medicine, Fujian Medical University Fuzhou China; 5 Cyberspace Institute of Advanced Technology, Guangzhou University Guangzhou China

**Keywords:** artificial neural network, college students, COVID-19, internet new media, logistic regression, machine learning, sleep quality

## Abstract

**Background:**

COVID-19 has been reported to affect the sleep quality of Chinese residents; however, the epidemic’s effects on the sleep quality of college students during closed-loop management remain unclear, and a screening tool is lacking.

**Objective:**

This study aimed to understand the sleep quality of college students in Fujian Province during the epidemic and determine sensitive variables, in order to develop an efficient prediction model for the early screening of sleep problems in college students.

**Methods:**

From April 5 to 16, 2022, a cross-sectional internet-based survey was conducted. The Pittsburgh Sleep Quality Index (PSQI) scale, a self-designed general data questionnaire, and the sleep quality influencing factor questionnaire were used to understand the sleep quality of respondents in the previous month. A chi-square test and a multivariate unconditioned logistic regression analysis were performed, and influencing factors obtained were applied to develop prediction models. The data were divided into a training-testing set (n=14,451, 70%) and an independent validation set (n=6194, 30%) by stratified sampling. Four models using logistic regression, an artificial neural network, random forest, and naïve Bayes were developed and validated.

**Results:**

In total, 20,645 subjects were included in this survey, with a mean global PSQI score of 6.02 (SD 3.112). The sleep disturbance rate was 28.9% (n=5972, defined as a global PSQI score >7 points). A total of 11 variables related to sleep quality were taken as parameters of the prediction models, including age, gender, residence, specialty, respiratory history, coffee consumption, stay up, long hours on the internet, sudden changes, fears of infection, and impatient closed-loop management. Among the generated models, the artificial neural network model proved to be the best, with an area under curve, accuracy, sensitivity, specificity, positive predictive value, and negative predictive value of 0.713, 73.52%, 25.51%, 92.58%, 57.71%, and 75.79%, respectively. It is noteworthy that the logistic regression, random forest, and naive Bayes models achieved high specificities of 94.41%, 94.77%, and 86.40%, respectively.

**Conclusions:**

The COVID-19 containment measures affected the sleep quality of college students on multiple levels, indicating that it is desiderate to provide targeted university management and social support. The artificial neural network model has presented excellent predictive efficiency and is favorable for implementing measures earlier in order to improve present conditions.

## Introduction

Since the outbreak of COVID-19, repeated outbreaks in various places have brought long-term and immeasurable impacts upon people's physical and mental health. In March 2022, a new round of cases broke out in Fujian Province, with Quanzhou, Putian, Ningde, and other cities involved. The epidemic strain was the Omicron variant (BA.2), which shows faster transmission speed and stronger immune escape ability. By April 21, a total of 3581 positive cases had been reported for the SARS-CoV-2 viral nucleic acid in Fujian Province. The Pittsburgh Sleep Quality Index (PSQI) questionnaire was released on the internet from April 5 to 16, 2022, and the scale was used to investigate the sleep quality of college students in Fujian Province in the past month. During this period, epidemic prevention and control were at a high platform stage, so there was still a risk of community transmission. Most colleges and universities in Fujian Province had comprehensively upgraded their epidemic prevention and control strategies and transferred to web-based teaching.

The existing literature has reported clearly that the epidemic will affect the sleep quality of Chinese residents, and the public exposed to stress events shows a variety of psychological problems, leading to sleep latency, increasing night wakefulness, reduced bedtime, and more sleep complaints generated [1-3]. Similarly to the 2003 severe acute respiratory syndrome outbreak in Beijing, the COVID-19 outbreak in Fujian became an acute, large-scale, and uncontrollable stressor, while the relevant negative information also led to irrational tension or fear [4]. Previous studies have shown that various types of mental health problems, including perceived stress and anxiety, are harmful to sleep quality, where stress is inversely correlated with sleep quality [5]. At the same time, mandatory self-isolation and closed prevention and control are not conducive to people to establish a healthy lifestyle [6]. Uncertainty during the developing situation of the epidemic may have caused fear and insecurity, and similar negative effects on mental health may deepen as the number of people with infection increases [7]. In addition, risk factors related to psychological problems during the epidemic control period included women, a history of previous mental illnesses, experiencing physical symptoms consistent with COVID-19, inadequate living with family members, concerns about relatives’ health, reduced social contact, lack of information, and economic losses. All these factors further affect people's sleep quality.

Sleep deprivation not only disrupts the circadian rhythm of the body and impairs the immune response, it also significantly decreases humans’ cognitive accuracy [8]. In severe cases, this leads to sleep disorders, increases the risk of infectious diseases, and affects the occurrence and progression of many other diseases, such as depression [9-11]. It is worth noting that young people’s sleep quality has a strong correlation between physical health and mental health [12]. Moreover, stressful college life greatly increases the demand for mental health care of college students. In the general population, college students seem to be particularly vulnerable to the negative effects of isolation.

Currently, more attention has been focused on the sleep condition of frontline health care workers, and older individuals and children in China; however, there are few reports on the impact of repeated epidemic situations and strict epidemic prevention and control measures on the sleep status of college students. Guaranteeing the mental health and sleep quality of college students, while preventing and controlling the epidemic, has become a thorny issue for college management. Machine learning (ML) has proven to be a powerful tool for sleep medicine research and has shown potential for improving prediction and visualization quality [13]. The successful development of models using ML has been widely applied to sleep stage classification, the automatic diagnosis of obstructive sleep apnea, etc [14,15].

Given that guaranteeing the mental health and sleep quality of college students while preventing and controlling the epidemic has become a thorny issue for college management, the aim of this study is to systematically investigate the sleep quality and potential predictors of college students in each city of Fujian under the repeated emergency of an epidemic. This study conducted a web-based questionnaire survey and evaluated the effect of strict epidemic prevention and control measures on college students’ sleep quality to explore the potential factors. Furthermore, this study developed prediction models for the classification of sleep quality among college students based on logistic regression (LR), artificial neural network (ANN), random forest (RF), and naive Bayes (NB) models, and compared performances among different models.

## Methods

### Data Sources

An internet-based cross-sectional survey was conducted from April 5 to 16, 2022, selecting full-time college students from 33 universities in Fujian as the study subjects, to assess their sleep quality. The questionnaire was compiled and issued using internet new media, a scientific research platform designed by experts from The Second Affiliated Hospital of Fujian Medical University. The self-designed platform ensures access, stability, data compatibility, and security by an upgraded system; creates a questionnaire with the vertical rolling design; and monitors the answer time to screen out the qualified questionnaire.

The inclusion criterion for this study was being a full-time college student in Fujian Province, undergraduate or graduate (aged 17-35 years). The exclusion criteria were as follows: (1) full-time undergraduate and graduate students who delayed in registering for a new semester due to the epidemic and lived in a place other than a student residence; and (2) people with major sleep disorders or major mental disorders.

Four items were covered in the survey, which were general personal information, sleep quality, learning and living conditions, and investigation of adherence. Participants self-reported related demographic variables (gender, age, college, major, study, height, weight, etc) and lifestyle variables (caffeine intake, staying up late, entertainment time, etc). Sleep quality was assessed using the PSQI scale [16]. The scale measures 7 domains composed of 18 self-assessment items, and the global score reflects the respondents’ sleep quality over the past month. Each component is graded using 0-3, and the global scores range from 0 to 21, with higher scores indicating poorer sleep quality. The Cronbach α value is .842, and the Chinese version was tested to have good reliability and validity [17].

The discrete and missing data were removed on the basis of demographic data indicators after the survey, and the analysis showed high internal consistency of related items (Cronbach α=.859).

### Statistical Analysis

A descriptive analysis was performed with all variables. Quantitative data consistent with a normal distribution were represented as means and SDs, while Student *t* test and ANOVA were applied. Chi-square test was used for qualitative data described as frequencies (percentages). To explore the influence of the severity of the current COVID-19 epidemic on factors related to sleep quality and the sleep disorder rate of college students in Fujian, this study successively conducted univariate and multivariate analyses. Factors with statistical significance in the univariate analysis were included in the multivariate nonconditional LR analysis. Multiple comparisons using ANOVA and the Bonferroni method were conducted to explore the differences in sleep quality among college students from different regions, with *P*<.001 in univariate analysis and *P*<.05 in multivariate analysis. A *P* value less than .05 was considered statistically significant. All statistical analyses were performed using SPSS (version 26; IBM Corp) and R (version 4.2.1; The R Foundation).

### Development and Validation of the Prediction Models

LR is a traditional model that is commonly applied to evaluate the contributions of independent variables and predict outcomes [14,15]. ANNs are emerging as a powerful algorithm for the prediction of medical diagnoses. An ANN model can be trained to recognize complex functional relationships between covariates and response variables. The literature has shown that ANNs are superior to linear models in several clinical fields [18,19]. RF is a typical supervised learning algorithm that is constructed from decision trees. The RF model predicts a class outcome using the majority vote of trees in order to minimize training error [20,21]. NB is a classification technique based on Bayes’ theorem. It is a simple model that is capable of working with noisy data and learning from small data sets, but it is not the ideal algorithm for high dimensionality problems with a large number of attributes [22,23].

**Figure 1:**
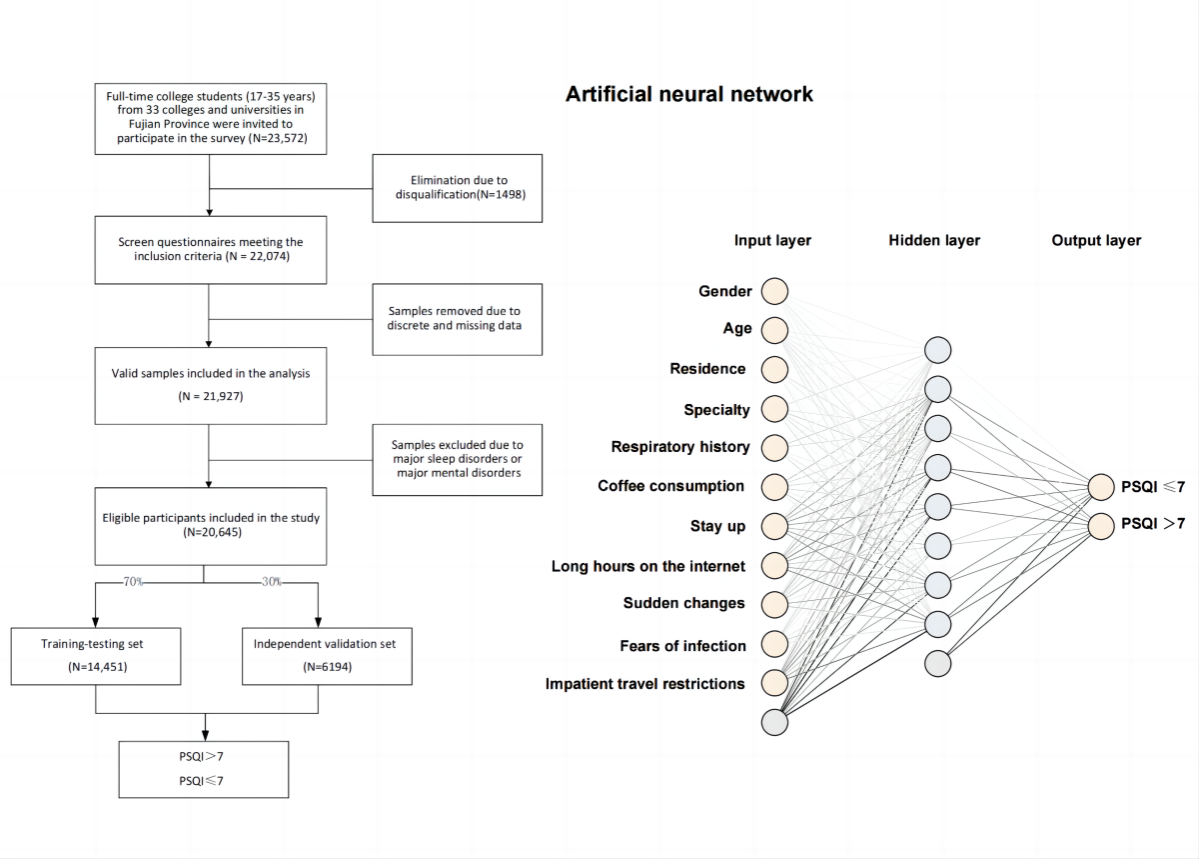
The study flowchart and artificial neural network (ANN) model. PSQI: Pittsburgh Sleep Quality Index.

A total of 4 models were developed using LR, ANN, RF, and NB. A PSQI score of 7 was used as the cutoff point to conduct sample grouping, with a score >7 defined as poor sleep and a score ≤7 defined as good sleep quality. All models were trained to predict the sleep quality of college students during the epidemic, using significant variables found to influence sleep quality (Figure 1).

The included data were partitioned into a training-testing set (n=14,451, 70%) and an independent validation set (n=6194, 30%). Stratified sampling based on sleep quality assessment results was conducted. The areas under curves (AUCs) of the 4 models in the training set were evaluated to assess model performance. AUC values closer to 1 indicate better performance. In addition, we calculated the accuracy, sensitivity, specificity, positive predictive value (PPV), negative predictive value (NPV), and other model performance indicators.

All models were established using R.

### Ethical Considerations

Ethical review in the data collection procedure was obtained from the Medical Ethics Committee of the Second Affiliated Hospital of Fujian Medical University, Sleep Medicine Key Laboratory of University in Fujian, and the Sleep Disorder Medicine Center of the Second Affiliated Hospital of Fujian Medical University (IRB No. 2021-309). The participants were informed of the purpose and confidentiality means of this anonymous research and were free to withdraw from the survey at any time without any reason. Responses to the questionnaire were considered as informed consent.

## Results

### Participant Characteristics

A total of 23,572 questionnaires were collected, including 20,645 valid questionnaires, with a response rate of 87.58% (n=20,645). Table 1 shows descriptive statistics of sample characteristics, including age, gender, residence, specialty, grade, and BMI.

**Table 1:** Characteristics of the respondents in this survey (N=20,645).

Characteristic		Participants, n (%)
**Age**		
	<20 years	9227 (44.7)
	≥20 years	11,418 (55.3)
**Gender**		
	Male	6326 (30.6)
	Female	14,319 (69.4)
**BMI**		
	<18.5	4804 (23.3)
	18.5-24	12,513 (60.6)
	24-28	2303 (11.2)
	≥28	1025 (4.9)
**Residence**		
	Quanzhou	4959 (24.9)
	Fuzhou	3782 (18.3)
	Longyan	7066 (34.2)
	Nanping	897 (4.3)
	Ningde	540 (2.6)
	Putian	190 (0.9)
	Sanming	2133 (10.3)
	Xiamen	437 (2.1)
	Zhangzhou	641 (3.1)
**Specialty**		
	Medical-related majors	4088 (19.8)
	Science and engineering	7876 (38.2)
	Liberal arts	8681 (42)
**Grade**		
	Graduating class	1459 (7.1)
	Nongraduating class	19,186 (92.9)

### Sleep Quality of College Students in Fujian Province During the COVID-19 Epidemic

The mean PSQI score of participants was 6.02 (SD 3.112), with scores ranging from 0 to 21. Higher scores indicated worse sleep quality. Statistical analysis showed that during the COVID-19 outbreak in Fujian, among the participants included in the sample, 14,673 college students had good sleep quality, while 5972 had poor sleep quality. The detection rate of sleep disturbance was 28.9% (n=5972).

Among these data, the mean score for males was 5.55 (SD 3.2), and the counterpart was 6.23 (SD 3.048). There were differences in the 7 domains of the PSQI among genders (Table 2). Thus, there were 7 domains in the global PSQI scores among college students in different cities in Fujian (*P*<.001). The results of the ANOVA are shown in Table 3.

**Table 2:** Comparison of the detection rate of 7 sleep problems among different genders in this survey. Each Pittsburgh Sleep Quality Index component score of >1 point indicates a sleep problem.

Variables	Subjective sleep quality	Sleep onset latency	Sleep duration	Sleep efficiency	Sleep disturbances	Use of sleep medications	Daytime dysfunction
Males, n (%)	1511 (23.9)	2301 (36.4)	1050 (16.6)	867 (13.7)	856 (13.5)	146 (2.3)	2572 (40.7)
Females, n (%)	3619 (25.3)	6343 (44.3)	2276 (15.9)	2590 (18.1)	2784 (19.4)	282 (2.0)	6623 (46.3)
Chi-square (*df*)	4.530 (3)	113.194 (3)	1.605 (3)	60.447 (3)	105.569 (3)	2.477 (3)	55.616 (3)
*P* value	.03	<.001	.21	<.001	<.001	.12	<.001

**Table 3:** Comparison of sleep quality scores of college students in different regions in this survey.

	Subjective sleep quality	Sleep onset latency	Sleep duration	Sleep efficiency	Sleep disturbances	Use of sleep medications	Daytime dysfunction	Global PSQI score
Quanzhou, mean (SD)	1.088 (0.752)	1.397 (0.982)	0.789 (0.741)	0.822 (0.899)	1.003 (0.628)	0.051 (0.308)	1.201 (0.970)	6.03 (3.205)
Fuzhou, mean (SD)	1.131 (0.763)	1.438 (0.992)	0.751 (0.736)	0.825 (0.881)	1.062 (0.6122^a^)	0.082 (0.399^a^)	1.457 (0.995^a^)	6.36 (3.171^a^)
Longyan, mean (SD)	1.013 (0.727^a^)	1.217 (0.923^a^)	0.959 (0.675^a^)	0.504 (0.745^a^)	0.997 (0.626)	0.068 (0.351)	1.369 (0.974^a^)	5.75 (2.977^a^)
Nanping, mean (SD)	0.979 (0.746^a^)	1.362 (1.001)	0.813 (0.715)	0.726 (0.872)	0.988 (0.643)	0.069 (0.386)	1.252 (0.949)	5.84 (3.243)
Ningde, mean (SD)	1.104 (0.751)	1.413 (1.002)	0.609 (0.706^a^)	0.902 (0.893)	0.976 (0.0589)	0.057 (0.353)	1.244 (0.945)	5.96 (3.098)
Putian, mean (SD)	0.979 (0.674)	1.126 (0.917^a^)	0.816 (0.645)	0.563 (0.730^a^)	0.937 (0.569)	0.047 (0.258)	1.226 (0.924)	5.33 (2.694)
Sanming, mean (SD)	1.063 (0.742)	1.386 (0.953)	1.008 (0.735^a^)	0.605 (0.825^a^)	1.052 (0.639)	0.094 (0.418^a^)	1.426 (0.966^a^)	6.23 (3.085)
Xiamen, mean (SD)	1.112 (0.666)	1.359 (0.922)	1.059 (0.743^a^)	0.595 (0.783^a^)	1.103 (0.587)	0.087 (0.436)	1.652 (0.945^a^)	6.53 (2.855^a^)
Zhangzhou, mean (SD)	1.103 (0.792)	1.454 (0.994)	0.786 (0.768)	0.908 (0.963)	1.087 (0.670^a^)	0.097 (0.464)	1.262 (1.025)	6.37 (3.353)
The global score, mean (SD)	1.063 (0.744)	1.339 (0.966)	0.865 (0.723)	0.685 (0.849)	1.020 (0.626)	0.070 (0.365)	1.344 (0.980)	6.02 (3.112)
*F* test (*df*)	10.996 (8, 20,636)	24.917 (8, 20,636)	59.255 (8, 20,636)	85.393 (8, 20,636)	7.398 (8, 20,636)	4.024 (8, 20,636)	30.118 (8, 20,636)	17.303 (8, 20,636)
*P* value	<.001	<.001	<.001	<.001	<.001	<.001	<.001	<.001

^a^Compared with Quanzhou, *P*＜.05.

### Analyses of Factors Influencing Sleep Quality

A univariate analysis was used to analyze influencing factors of sleep quality on college students. The results of the chi-square test showed that during the local COVID-19 outbreak in Fujian Province in 2022, the sleep quality of college students was correlated with following 12 variables: age, gender, grade, residence, specialty, respiratory history, coffee consumption, stay up, long hours on the internet, sudden changes, fears of infection, and impatient closed-loop management (*P*<.001), but this relationship appears to be independent of BMI.

A multivariate unconditional LR analysis was applied to determine predictors of the sleep quality of college students during the epidemic. The PSQI score was selected as the dependent variable (poor sleep quality=1, good sleep quality=0). The 12 variables that showed statistical significance in univariate analysis were included in multivariate nonconditional LR analysis as independent variables. Results showed that a total of 11 variables were related to sleep quality, which were age, gender, residence, specialty, respiratory history, coffee consumption, stay up, long hours on the internet, sudden changes, fears of infection, and impatient closed-loop management. Details are presented in Table 4.

**Table 4:** Analysis of factors affecting sleep quality (number) among college students in this survey.

Variables		Participants (N=20,645), n	Sleep quality, n (%)		Univariate analysis		Multivariate analysis, OR^a^ (95% CI)	*P* value
			Good (≤7)	Poor (>7)	Chi-square (*df*)	*P* value		
**Age**					64.732 (1)	<.001		<.001
	＜20	9227	6819 (46.5)	2408 (40.3)			1 (reference)	
	≥20	11,418	7854 (53.5)	3564 (59.7)			1.31 (1.23-1.40)	
**Gender**					69.801 (1)	<.001		<.001
	Male	6326	4747 (32.4)	1579 (26.4)			1 (reference)	
	Female	14,319	9926 (67.6)	4393 (73.6)			1.14 (1.06-1.23)	
**Residence**					114.574 (8)	<.001		
	Quanzhou	4959	3502 (23.9)	1457 (24.4)			1 (reference)	N/A^b^
	Fuzhou	3782	2519 (17.2)	1263 (21.1)			0.92 (0.83-1.02)	.11
	Longyan	7066	5293 (36.1)	1773 (29.7)			0.71 (0.64-0.78)	<.001
	Nanping	897	655 (4.5)	242 (4.1)			0.91 (0.77-1.09)	.31
	Ningde	540	392 (2.7)	148 (2.5)			0.83 (0.67-1.03)	.09
	Putian	190	146 (1)	44 (0.7)			0.78 (0.54-1.11)	.17
	Sanming	2133	1451 (9.9)	682 (11.4)			0.93 (0.82-1.06)	.29
	Xiamen	437	291 (2)	146 (2.4)			0.93 (0.74-1.16)	.52
	Zhangzhou	641	424 (2.9)	217 (3.6)			1.57 (1.29-1.9)	<.001
**Specialty**					163.534 (2)	＜.001		
	Medical-related majors	4088	3120 (21.3)	968 (16.2)			1 (reference)	N/A
	Science and engineering	7876	5780 (39.4)	2096 (35.1)			1.42 (1.27-1.58)	<.001
	Liberal arts	8681	5773 (39.3)	2908 (48.7)			1.62 (1.46-1.8)	<.001
**Grade**					5.443 (1)	.02		.63
	Graduating class	1459	998 (6.8)	461 (7.7)			1 (reference)	
	Nongraduating class	19,186	13,675 (92.3)	5511 (92.3)			1.03 (0.91-1.17)	
**BMI**					5.127 (3)	.16	—^c^	—
	<18.5	4804	3381 (23)	1423 (23.8)			—	—
	18--5,24	12,513	8960 (61.1)	3553 (59.5)			—	—
	24-28	2303	1604 (10.9)	699 (11.7)			—	—
	≥28	1025	728 (5)	297 (5)			—	—
**Respiratory history**					146.882 (1)	＜.001		<.001
	No	15,477	11,342 (77.3)	4135 (69.2)			1 (reference)	
	Yes	5168	3331 (22.7)	1837 (30.8)			1.35 (1.25-1.45)	
**Coffee consumption**					415.938 (3)	<.001		
	No	10,290	7884 (53.7)	2406 (40.3)			1 (reference)	N/A
	Occasionally	8331	5652 (38.5)	2679 (44.9)			1.18 (1.10-1.27)	<.001
	Often	1441	799 (5.4)	642 (10.8)			1.55 (1.37-1.76)	<.001
	Almost everyday	583	338 (2.3)	245 (4.1)			1.29 (1.07-1.56)	.007
**Stay up**					780.661 (3)	<.001		
	Not matched	10,951	8519 (58.1)	2432 (40.7)			1 (reference)	N/A
	Sometimes matched	7738	5195 (35.4)	2543 (42.6)			1.35 (1.26-1.45)	<.001
	Often matched	1442	746 (5.1)	696 (11.7)			1.93 (1.71-2.18)	<.001
	Always matched	514	213 (1.5)	301 (5)			2.24 (1.84-2.73)	<.001
**Long hours on the internet**					986.397 (3)	<.001		
	Not matched	5892	4838 (33)	1054 (17.6)			1 (reference)	N/A
	Sometimes matched	8915	6483 (44.2)	2432 (40.7)			1.29 (1.18-1.41)	<.001
	Often matched	4005	2457 (16.7)	1548 (25.9)			1.83 (1.66-2.03)	<.001
	Always matched	1833	895 (6.1)	938 (15.7)			2.67 (2.36-3.02)	<.001
**Sudden changes**					117 (1)	<.001		<.001
	No	19,792	14,207 (96.8)	5585 (93.5)			1 (reference)	
	Yes	853	466 (3.2)	387 (6.5)			1.89 (1.63-2.2)	
**Fears of infection**					120.260 (3)	<.001		
	Not matched	8038	5922 (40.4)	2116 (35.4)			1 (reference)	N/A
	Sometimes matched	10,274	7302 (49.8)	2972 (49.8)			0.98 (0.92-1.06)	.64
	Often matched	1673	1055 (7.2)	618 (10.3)			1.19 (1.05-1.34)	.005
	Always matched	660	394 (2.7)	266 (4.5)			1.25 (1.04-1.49)	.02
**Impatient closed-loop management**					1037.383 (3)	<.001		
	Not matched	7663	6250 (42.6)	1413 (23.7)			1 (reference)	N/A
	Sometimes matched	8944	6252 (42.6)	2692 (45.1)			1.59 (1.47-1.72)	<.001
	Often matched	2252	1293 (8.8)	959 (16.1)			2.4 (2.15-2.67)	<.001
	Always matched	1786	878 (6)	908 (15.2)			3.06 (2.72-3.45)	<.001

^a^OR: odds ratio.

^b^N/A: not applicable.

^c^Not determined.

### Model Performance

**Figure 2:**
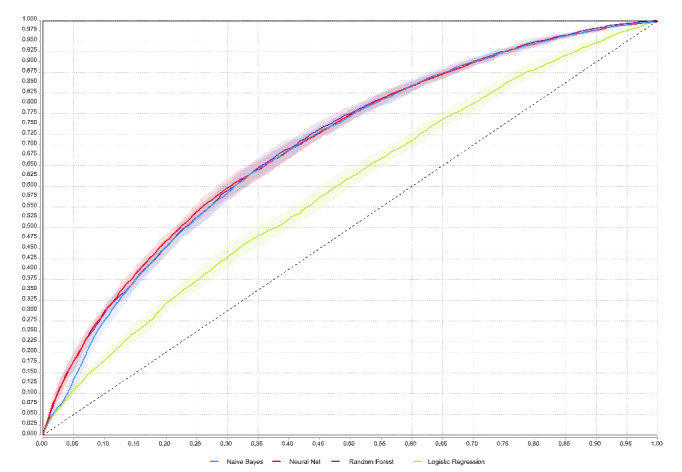
Areas under curves (AUCs) of the 4 models developed.

As mentioned previously, there were 11 input parameters for the prediction models for screening college students with poor sleep quality. Following adequate training, the 4 models were applied to the validation set. Table 5 shows metrics of all models. The generated models all revealed better model performance, except for the LR model, which had the lowest AUC of 0.59. The ANN model achieved the highest AUC of 0.713, with accuracy, sensitivity, specificity, PPV, and NPV of 73.52%, 25.51%, 92.58%, 57.71%, and 75.79%, respectively. The NB model showed a significant and high sensitivity. Figure 2 shows the AUC of the 4 models applied to the validation set.

**Table 5:** Model performance metrics of 4 models.

Characteristic	Logistic regression	ANN^a^	Random forest	Naive Bayes
AUC^b^	0.59	0.713	0.708	0.707
Kappa	0.079	0.218	0.167	0.236
Delta-P	0.183	0.335	0.329	0.275
Youden index	0.061	0.181	0.132	0.214
Accuracy, %	70.91	73.52	73.09	71.8
Sensitivity, %	11.7	25.51	18.47	35
Specificity, %	94.41	92.58	94.77	86.4
Positive predictive value, %	45.37	57.71	58.35	50.53
Negative predictive value, %	72.93	75.79	74.54	77.01
Positive likelihood ratio	2.093	3.438	3.532	2.574
Negative likelihood ratio	0.935	0.805	0.86	0.752
Lift, %	159.69	203.11	205.35	177.84
Fallout, %	5.59	7.42	5.23	13.6
F measure, %	18.61	35.38	28.05	41.36
Classification error, %	29.09	26.48	26.91	28.2

^a^ANN: artificial neural network.

^b^AUC: area under curve.

## Discussion

### Principal Findings

This study found that about 28.9% (n=5972) of college students represented sleep disturbances, which indicated that the COVID-19 pandemic was a risk factor. This result is consistent with a horizontal study by Duan et al [24], which showed that the COVID-19 pandemic led to sleep disorders among college students in Wuhan.

Sleep is the most basic physiological need of human beings, and good sleep quality contributes to better mental health, serving as a foundation for a healthy life and efficient learning for college students [25,26]. College students with poor sleep require interventions. ML methods have been widely applied to predict sleep disorders; however, prediction models for screening poor sleep quality in the context of COVID-19 prevention with eligible predictors are insufficient.

This study has developed and validated prediction models for self-reported variables, which may contribute to early screening of sleep problems in college students. The ANN model trained with 11 parameters as input exhibited excellent predictive power, achieving an AUC of 0.713. A previous study using the decision tree, RF, support vector machine, ANN, and gradient boosting trees to assess the prevalence of sleep disturbances among university students found that the best model was the RF model, which obtained 74% accuracy and 95% specificity [27]. This result is close to our research.

According to Table 4, the related factors that affected the sleep quality of college students included age, gender, residence, specialty, respiratory history, coffee consumption, stay up, long hours on the internet, sudden changes, fears of infection, and impatient closed-loop management. The prevalence rate of sleep disturbance in women (n=4393, 30.7%) was significantly higher than that in men (n=1579, 25%), and the difference was statistically significant (*P*<.001), which is consistent with earlier findings [28]. Medical students were found to have a better sleep quality, a result that was in line with that of a previous study by Yang et al [29]. The findings in Table 4 indicate that daily caffeine intake and sleep quality are inversely correlated. The tendency to stay up late to complete learning due to closed-loop management is a risk factor for sleep disorders, and this risk increases with the frequency of staying up late. Increased electronic equipment entertainment time is positively correlated with decreased sleep quality. The risk of sleep disturbance in those who felt agitated and depressed due to strict epidemic prevention and control management was 3.08 times higher than that of “no irritability.”

Previous studies have verified the association between these predictors and sleep quality. In addition to uncontrollable factors, such as being far away from home, behavioral factors, including sleep schedule, screen time, exercise, and diet, have been reported to be the main influencing factors responsible for poor sleep in college students [30,31]. Sawah et al [32] and Lohsoonthorn et al [33] found that caffeine intake affected evening sleep quality in medical students and that the intake was inversely associated with sleep quality. The outbreak of COVID-19 and the following prevention and control measures were stressors that may have induced negative emotions and indicated the decline in sleep quality [34].

In this context, this study used cross-sectional survey data to develop predictive models. LR, ANN, RF, and NB are common modeling tools in biomedicine. The LR model underperformed in the validation set among the generated models, which may have been related to our data structure. When the predictor-outcome relationships in a data set are relatively complex and nonlinear, LR may achieve unsatisfactory performance. ANN have outstanding ability to detect nonpredefined relations, such as nonlinear effects and interactions [35]. This may be related to the reason the ANN model achieved the best predictive validity in predicting sleep disturbances among university students.

With prominent specificity but low sensitivity, there was a very low probability for the established prediction models to classify respondents who did not actually have sleep disorders as those with sleep disturbance, indicating that a sleep disorder is highly suspected if the result is positive. This advantage is conducive to picking out individuals with poor sleep quality and focusing on them. Therefore, the generated models are beneficial for decision makers to understand the sleep status of college students under closed-loop management of the epidemic, and to promote the diagnosis of sleep disorders. However, this kind of model may lead to a large number of missed diagnoses in very large-scale screening. Attention should be paid when using the prediction model. On the basis of the existing prediction model, the predictive variables can be changed to further improve the prediction ability of the model. For example, subjective sleep factors such as sleep duration and difficulty falling asleep can be introduced. Furthermore, the introduction of new data sets from external cohort populations may be considered for external validation of the model.

This study defined poor sleep as a PSQI score >7. If the cutoff point is set to a lower level (eg, 5) [36], there is a probability for the model to detect more positive samples and increase its sensitivity. However, this may result in a number of true negative samples being misjudged as positive, hence reducing the specificity of the model accordingly, and the AUC value may also change. Therefore, it is crucial for an effective prediction model to find the optimal cutoff value for differentiating between individuals. In addition, other ML models or fusion models may be applied to achieve better performance for further studies.

### Strengths and Limitations

This cross-sectional study was conducted through the internet, which is conducive to data collection with a large sample size and extensive geographic coverage. The respondents were recruited from 9 cities in Fujian, with strong representativeness. There was relatively little heterogeneity in the control measures in various colleges and universities in the province, and the results were good in general. The collected data covered the assessment of sleep quality, factors of prevention and control measures, lifestyle and individual factors, etc. Our study still had several limitations. The strengths and weaknesses of this study have been summarized in Textbox 1.

**Pros and cons of the existing research work.**

Strengths:

- Large sample size
- High-quality data with strong representativeness
- The study has strong timeliness
- Comprehensive items were investigated
- Build prediction model based on machine learning algorithms

Limitations:

- The causal relationship of the research objects remains unknown
- The data source is subjective and lacks objective measurement
- Potential selection bias and nonresponse bias
- More factors affecting sleep quality should be studied
- The effectiveness of residence in the prediction model should be verified

First, the causal relationship between the strict control measures after the COVID-19 outbreak and the sleep quality of college students cannot be determined through cross-sectional studies alone. In addition, the characteristics of the cross-sectional study and the assessment scope of the PSQI scale make it difficult to explore the association between different levels of epidemic prevention and control measures and the sleep quality of college students.

Second, the data were self-reported by respondents, and subjective sleep reporting may overestimate or underestimate sleep quality. Despite the commitment to confidentiality, the results may still be influenced by social desirability bias, leading to possible reporting bias. Some studies have incorporated objective data into prediction models. Zhang et al [37] developed a back-propagation ANN prediction model, taking objective data measured through physical examination and subjective data collected through questionnaires as input.

Third, there may have been selection bias in this internet-based survey, since college students who are relatively more active on social media were more likely to respond, while students with insufficient recognition and attention toward sleep health were more inclined not to respond to this study, resulting in nonresponse bias. This may also have led to generally longer screen times for the respondents.

Fourth, considering the patience of college students in the survey process and the richness of the questionnaire content, this study did not investigate excessive relevant factors affecting sleep quality, such as exercise; thus, it is difficult to ensure the highest quality of the questionnaire.

Finally, the effectiveness of residence in the prediction model was unclear. Previous studies have shown that the epidemic severity for those living in the province near the epicenter was not significantly associated with mental health problems. Regardless of the epidemic severity, acute stress, anxiety, and depression are pervasive in Chinese college students [38], and that kind of psychological distress may affect sleep quality.

### Future Research

Future studies are needed to use an experimental or longitudinal design combined with followed-up visits to explore issues more in depth. Key variables of the prediction model could apply objective indicators, which can be evaluated through observation and objective measurement. Furthermore, other significant predictors of sleep quality could be explored, and wearable devices could also be used as a means of assessing sleep.

### Conclusions and Recommendations

Despite several limitations, there is sufficient evidence in this study to show that the upgrade in epidemic prevention and control management caused by the local outbreak in Fujian Province had a negative impact on the sleep status of college students. About 28.9% (n=5972) of college students reported poor sleep quality. A total of 11 variables were found to be related to sleep quality, including staying up, fears of infection, and impatient closed-loop management. These findings highlight the need for a targeted measure to improve sleep status of college students during the COVID-19 outbreaks. The ANN model globally represented the strongest predictive ability for sleep quality of college students, with an AUC, accuracy, sensitivity, specificity, PPV, and NPV of 0.713, 73.52%, 25.51%, 92.58%, 57.71%, and 75.79%, respectively. The results show the prospect of using ML techniques to predict college students’ sleep quality with only a few key variables collected, providing strong data and tool support for corresponding intervention and prevention strategies.

## Abbreviations

ANN: artificial neural network
AUC: area under curve
LR: logistic regression
ML: machine learning
NB: naive Bayes
NPV: negative predictive value
PPV: positive predictive value
PSQI: Pittsburgh Sleep Quality Index
RF: random forest

## Data Availability

The source code and the data sets used in this study are freely available on GitHub [39].
